# Vulnerable exposures and outcomes for children of female sex workers in low- and middle-income countries: application of the bioecological vulnerability framework

**DOI:** 10.3389/fpubh.2025.1642053

**Published:** 2025-09-24

**Authors:** Swarna D. S. Weerasinghe, Meghan Fitzgerald, Emily K. Perttu, Brian Wills, Wendy L. Macias-Konstantopoulos

**Affiliations:** ^1^Department of Community Health and Epidemiology, Dalhousie University, Halifax, NS, Canada; ^2^Global Health Promise, Portland, OR, United States; ^3^Center for Social Justice and Health Equity, Department of Emergency Medicine, Massachusetts General Hospital, Harvard Medical School, Boston, MA, United States

**Keywords:** children of female sex workers, vulnerability assessment, child and adolescent health, developmental exposures and outcomes, bioecological model of development

## Abstract

**Introduction:**

Children of female sex workers (CFSW) are exposed to unique bioecological vulnerabilities that negatively affect their developmental outcomes. We interviewed 1,280 female sex worker mothers (FSWM) in eight low- and middle-income countries (Angola, Brazil, South Africa, the Democratic Republic of Congo, India, Indonesia, Kenya, and Nigeria) between January 16 and October 1, 2019. This exploratory study focused on multilayered exposures from early childhood to adolescence, resulting in maldevelopment and other negative outcomes for CFSW, as reported by their mothers.

**Methods:**

We used Bronfenbrenner’s revised *Bioecological System* Theory of *Human Development* as the analytical framework. The interview data included both numerical responses and brief textual answers. Quantitative information was summarized using prevalence estimates, while textual responses were inductively coded and grouped into major categories.

**Results:**

FSWM reported that outcomes varied across countries but reflected complex interactions within and between the micro-, meso-, exo-, and macro-systems described by the theoretical framework. Findings revealed that CFSW were exposed to suboptimal child-rearing environments and faced a high prevalence of adverse outcomes. A majority (61%; country range, 45–67%) of daughters entered sex work at an early age (mean age: 14 years; range, 13.5–18 years). Both sons and daughters experienced physical harm (72%; range, 21–85%) and sexual abuse (sons, 57%; daughters, 74%). Daughters were often introduced to sex work as a survival strategy. Reported outcomes also highlighted bioecological vulnerabilities that impeded sons’ development, as well as protective environments that supported their healthy growth. These were categorized as follows: (1) vulnerabilities associated with high-risk living environments such as brothels, hotspots, and streets where they were exposed to sex work; (2) negative psychological, physical, and behavioral outcomes, including becoming criminals, victims, and perpetrators of abuse; and (3) protective environments of living away from the mother’s work and finding informal work that may mitigate the harmful effects of bioecological vulnerable exposures.

**Discussion:**

The CFSW experienced significant threats to healthy development, contributing to adverse physical and psychological developmental outcomes. These findings underscore the urgent need for evidence-based policy directives, interventions, and support.

## Introduction

Globally, the majority (up to 90%) of female sex workers in low- and middle-income countries (LMICs) are mothers with more than three children on average (1–3). Less is known about the vulnerabilities of the children of female sex workers (CFSW) due to difficulties in accessing this hidden population (1). Several studies indicate that female sex workers who are mothers (FSWM) are often single parents and struggle to manage the dual roles of sex work and motherhood (2–4). Many FSWM raise children, hoping for future prosperity (5), but struggle to provide the necessary security and safety in their living environments (4). This study explored female sex workers’ sons’ and daughters’ vulnerabilities from a bioecological environmental exposure perspective. We explored how various exposures impacted their physical and psychological development, using data gathered from FSWMs in eight LMICs.

Child vulnerability is conceptualized as a relative state in which a child is exposed to greater physical and psychological risks than peers, such as lacking proper parental care, food, or education, and being subject to exploitation, abuse, neglect, violence, or exposure to disease, including HIV infection (6). The Organization for Economic Cooperation and Development conceptualizes child vulnerability as the manifestation of a combination of individual, familial, and social-environmental conditions (7), which child development researchers conceptualize as bio-ecological vulnerabilities (8). Impacts of FSWM child vulnerabilities are noted in the literature. Studies have suggested that FSWM living in high-risk environments experience multi-generational trauma that negatively impacts their children’s psychological and physical development (9). FSWM are engaged in child vulnerability reduction measures, such as living and working apart from children, to conceal their occupational identity from their children, and their children are raised by other family members (10). The United Nations Convention on the Rights of the Child recommends that children be raised in a nurturing environment by their mothers (1, 11), but FSWM are often unable to provide these environments (10). Our study examined the impact on CFSW development and well-being resulting from environmental exposures that affect vulnerable child-rearing and living conditions.

Vulnerabilities specific to CFSW are often associated with sex work and related social, economic, and environmental exposures (12). Child vulnerability can be fluid and context-dependent and neither well-defined nor thoroughly analyzed; however, the sources of vulnerability can be situational or inherent (13). Children of female sex workers often live in populations at higher risk for contracting HIV, including drug users and sex workers. CFSW experience elevated vulnerability in all areas of development, care, and protection, including food insecurity and malnutrition, low access to essential health services, reduced school enrollment, inadequate childcare, and exposure to physical and sexual violence (1, 14, 15).

Negative outcomes of vulnerable exposures among CFSW living in high-risk environments, such as living with an HIV-infected mother or a mother who uses substances, include truancy-related psychosocial issues, early sexual debut, introduction to sex work during adolescence, and social marginalization (16–18). All these outcomes are considered negative developmental outcomes. One study found that brothel-based children in Mumbai, India, experienced considerable health and developmental challenges despite their mothers’ efforts to keep them protected, educated, and employed (19). Another study found a higher prevalence of childhood victimization among Ugandan adolescent CFSW compared to their non-CFSW counterparts (9).

The current study is the first multi-LMIC country, community-based study of developmental outcomes for CFSW in the FSWM. Although previous research identified a higher risk of sexual victimization for daughters and sons of FSWM and the sexual exploitation of underage daughters (9, 20, 21), little is known about physical and psychological developmental outcomes for CFSW resulting from vulnerable exposures.

This research explored the bioecological vulnerability related to physical and psychological health development outcomes of CFSW raised in a sex work environment through the encounters and experiences of FSWM in eight LMIC. One aim of the study was to produce findings that can inform the targeting of multisectoral interventions and services for CFSW. Many of these services have been found to be effective in protecting the safety of CFSW and reducing victimization (9). Many services are delivered by non-governmental organizations and include peer support components that effectively contribute to the well-being of CFSW (12).

## Conceptual framework

**Figure 1:**
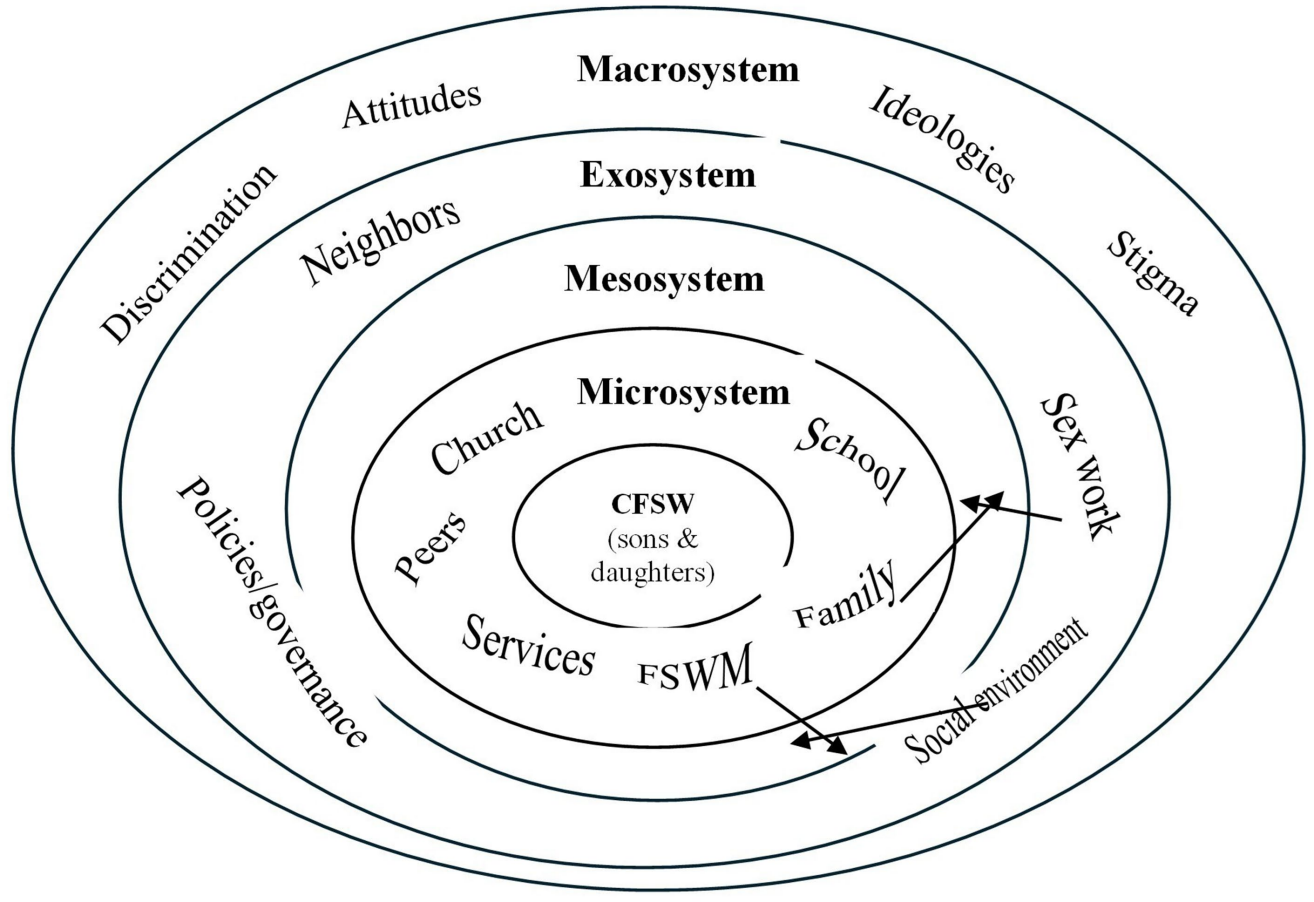
Working model of CSFW bioecological exposure explorative: Adapted from the Bronfenbrenner’s ecological model of human development revised version (22). Arrow above interactivity of the bioecological exposure.

Our analytical frameworks, including the interpretation of results, were guided by Bronfenbrenner’s revised bioecological model of human development (22). This model provides a theory of human development that postulates a child’s physical and psychological development, as well as the influence of a complex system of interconnected relationships, which occurs in four layers: microsystem, mesosystem, exosystem, and macrosystem. The working model depicting influences on CFSW, based on Bronfenbrenner’s theoretical framework (23), is shown in Figure 1. In our model, relationships exist in children’s immediate environments, in the most proximal concentric circle of influence that includes sex work (microsystem), and in the broader structural ecosystem of political, economic, social, and cultural influences of the society the child inhabits, depicted in the outermost concentric circle (macrosystem). This bioecological system of CFSW exposure encompasses the investigation of both negative and positive outcomes resulting from unfavorable and favorable exposures. The components of the working model (Figure 1) were informed by the literature. We quantitatively explored three outcomes specifically to address (a) daughters’ “entering sex work,” (b) physical harm experiences of sons and daughters; (c) sexual abuse experiences of sons and daughters; and a fourth outcome of (d) the developmental outcomes for FSW sons, with a detailed exploration of FSW daughters to a lesser extent. Part (d) was explored qualitatively using coding and categorization. The emphasis of the research areas was guided by the community partners.

The current study is the first multi-country LMIC study to explore FSWM descriptions of what happened to their children and children of other sex workers known to them. Studies that have explored parental encounters of children’s vulnerability to emotional ill health and behavioral outcomes suggest respondents needed to be closely associated with the child to understand the experiences (24). FSWM are as knowledgeable about other CFSWs in the community as they are about their own. One study demonstrated that close relatives can accurately assess a child’s quality of life, while another suggested that proxy respondents, such as parents, should be closely associated with the child and share the same values, priorities, and preferences as the child (24). Though our study participants were mothers in sex work, they may not only be biological mothers, as many children in high-poverty communities live with non-biological parents (23).

## Materials and methods

The current study is a cross-sectional, pilot, exploratory descriptive study that employed quantitative (numerical) and qualitative (text) data collection and analysis. This study explored vulnerable exposures that impede the physical and psychological development of their own children and the children of other sex workers in their geographic community.

### Study setting

Study country selection criteria included: (1) a high number of female sex workers (FSW); (2) a high number of maternal deaths; (3) high HIV rates among FSW; (4) established partnerships with local sex worker organizations (SWOs) and NGOs providing services to FSW; and (5) geographic regional representation. Eight LMICs were selected: Angola, Brazil, South Africa, Democratic Republic of the Congo (DRC), India, Indonesia, Kenya, and Nigeria. An initial list of potential study countries was narrowed based on the NGOs’ responses to our initial invitation to participate in the study.

### Participant recruitment

FSWM (study participants) were recruited through purposive sampling conducted by local partners. Participants were recruited in locations where the partners worked, including bars, brothels, parks, and fields, and were screened for eligibility. NGO partners applied the following inclusion criteria: (1) women aged≥18 years; (2) mothers of at least one child aged 10 years or younger; (3) engaged in full-time sex work during the past 3 years; and (4) socially interactive with other FSWM, familiar with their children, and living within the same communities. To maintain confidentiality and allow participants to discuss sensitive issues without concern for their own identity or the identities of their children and other FSWM being disclosed, demographic data were not collected. Informed consent was obtained from participants with either an “x” or a check mark on the consent form.

### Data collection

Data collection took place between 16th of January 2019 and October 1, 2019. Women shared knowledge from the encounters and experiences of their own children and other CFSWs in their community (25). Data were collected from 1,280 study participants in 24 cities, via 165 group meetings, across eight LMICs. The cities included Kenya (Nairobi, Mombasa, and Kisumu), Nigeria (Lagos, Calabar, and Abuja), DRC (Bukavu and Kinshasa), South Africa (Cape Town, Johannesburg, Durban, and Port Shepstone), India (Bangalore, Chennai, Salem, Nashik, Hyderabad, Warangal, and Gauribidanur), Indonesia (Jakarta), Brazil (Rio de Janeiro, Salvador, and São Paulo) and Angola (Luanda).

The research team collected responses to the following set of questions (Table 1) to characterize the vulnerable exposures and outcomes of CFSW based on the experiences and encounters of FSWM in the community, as well as their knowledge of other CFSW in their community, if not their own. The format of the questions (a numerical scale of 1 to 10) was agreed upon by community organizations to accommodate the study participants’ numeracy and literacy levels.

**Table 1:** Data collection instrument and measures.

Question	Measurement of vulnerability assessment	Scale (Type)
1. Where do FSWs in this community leave their children while working?	Early childhood exposure	Categorical: Alone, with family, crèche, and other (specify)
2a. Out of ten daughters of FSWM in this community, how many enter sex work?	Female child-specific exposure	Numerical scale of 1 to 10.
2b. At what age do daughters of FSWM “enter sex work”?	Origin of the female child: early exposure assessment	Numerical value.
3a. *Out of ten CFSW in the community, how many experience physical harm?	Physical vulnerability outcome	Numerical scale of 1 to 10
3b. *Out of ten CFSW in the community, how many experience sexual abuse?	Sexual vulnerability outcome	Numerical scale of 1 to 10
4. What happens to the sons of FSWM in the community?	Male-specific vulnerability detailed assessment	Text data

*Gender-specific questions were asked only in some countries.

During the study, the term “enter sex work” was used, as that is the terminology used by the study participants. We recognize that children, defined by the UN Convention on the Rights of the Child as any person under age 18, cannot consent to engaging in sex work. Where underage children are in the situation of entering sex work, it is considered sexual exploitation and sex trafficking. As these terms, especially the latter, have specific legal meanings under the laws of each country, both terms may apply; however, the legal implications differ depending on the country.

In this exploratory study, the data collection instrument (Table 1) evolved as the study progressed. Initial questions were formulated by researchers, guided by community partners, and informed by the researchers’ prior knowledge of the female sex worker community. The follow-up to question three (Tables 1, 3b) was asked in three countries: Brazil, Kenya, and South Africa, where data collection began. Due to participants offering varying responses about sons versus daughters in Kenya and Nigeria, this question evolved into two separate questions, one specific to sons and the other two daughters. This question was asked in the same manner in the remaining five countries. Angola’s responses to questions 2–4 (Table 1) were sparse and therefore excluded from the detailed analysis. We included an open-ended question (Question 4, Table 1) to explore specific outcomes for sons, given the lack of prior research-based evidence on sons’ outcomes.

Individual responses to question four were manually recorded but were not linked to a specific participant. Multiple responses from each participant were recorded as separate responses for data coding. Some participants commented on the outcomes for daughters of FSWM in response to question four; however, these comments were not substantive enough to support thematic coding and categorization. Some quoted responses for the daughter to question four are presented under the daughters of FSW. Angola was not included in the analysis of question four, since only one response was received.

All participants were allowed to respond to each question, and each participant responded only once. The lead researcher (BW) hand-recorded responses (audio recordings were not obtained with the consent of community partners) in South Africa, Kenya, and Nigeria, while translators assisted in collecting responses in Brazil, Angola, Indonesia, India, and the DRC. Details of the data collection methodology are published elsewhere (25).

### Data analysis

Community partners were not involved in the data analysis. The data consisted of both numerical and textual responses (Table 1). The research team summarized numerical responses using percentages, by country, for each category to provide a sense of the distribution across countries (questions 1–3, Table 1) and to provide country-level bioecological vulnerability characterizations. The average response for each country was calculated and then converted to a percentage by summing the individual Likert scale responses, which ranged from 1 to 10. The category-specific weighted average was calculated accounting for the number of study participants in each country (Sum [(country value) x (country average)]/ (total number participated)). Statistical inferences were not performed due to (a) purposive (non-probability) small sample selection; (b) questions about sexual abuse were not asked in all countries, and (c) the number of participants varied between countries.

### Rigor and trustworthiness of data collection and analysis

Though the research did not strictly follow community-based participatory research (CBPR) principles, the lead researcher (BW) engaged SWOs that provide support to FSW and, where there were no SWOs, with local NGOs that provide services to FSW as community partners. These local partners reviewed and approved the data collection questionnaires and assisted with data collection where translation was required. The lead researcher (BW) trained and supervised translators from local communities in Brazil, Angola, Indonesia, India, and the DRC, and in South Africa, Kenya, and Nigeria. The lead researcher administered the questions and recorded responses. Local partners determined the cities for data collection based on the study population’s geographic locations and to intentionally include both urban and rural settings. Next, local partners determined the venue for data collection in each city. Individual data collection within a group was recommended by local partners for safety reasons. Locations were selected based on the need for concealment of identity and the convenience and safety of both the participants (FSWM) and the research team.

Participants’ responses to open-ended question four (Table 1) were analyzed using free coding to determine major categories. The first and second authors (MF, who manually coded, and SW, who used text analytics) coded the comments independently and grouped them into broad categories. They then met to compare assigned codes and resolved any discrepancies with the lead researcher (BW), who had collected the data. This iterative process continued until an appropriate coding consensus and categorization were reached, a final set of codes and categories was agreed upon, and definitions for codes and categories were documented and then aligned with the working model (Figure 1). Direct quotes from the comments that further illustrated or emphasized categories are included in the results section. Although we did not conduct a formal data triangulation exercise, detailed text responses regarding what happened to sons, and to a lesser extent, what happened to daughters, were triangulated using quantitative responses in the results section.

### Ethics approval

The study protocol, informed consent forms, and data collection tools were reviewed and approved by the Institutional Review Board at Portland State University in Portland, Oregon, USA (Protocol #184888). Local partners reviewed the study protocol and data collection instruments to ensure compliance with local standards and approved the questionnaires and study procedures.

## Results

No demographic data were collected from the study participants or the CFSW; therefore, the research team summarized the quantitative responses to vulnerable exposures and outcomes first (responses to Table 1 questions 2 (a,b) and 3 (a,b)) to provide an overview of the children’s situation as a precursor to the detailed discussion that follows. In Table 2, we summarize the locations where mothers left their children (answers to Table 1, question 1), indicating early childhood vulnerability exposure. This question relates to the initial microsystem environmental exposures (Figure 1) that may help contextualize the structural determinants of negative health outcomes. Next, in the results section, a brief description of what happened to the daughters is provided, followed by a detailed analysis of what happened to the sons. The study participants’ descriptions of what happened to their daughters were consistent with the existing literature. The authors coordinated the detailed analysis of what happens to sons with the working model (Figure 1) to address gaps in the literature.

**Table 2:** Distribution of prevalence of negative outcomes for CFSW and child care practices.

	Nigeria	DRC	India	South Africa	Kenya	Indonesia	Brazil	All countries
	Average % (*n*)	Average (*n*)	Average % (*n*)	Average % (*n*)	Average % (*n*)	Average % (*n*)	Average % (*n*)	Weighted average %
Where FSWM leaves children, while at work								
Alone	13 (33)	37 (101)	0 (0)	2 (1)	19 (65)	0 (0)	0 (0)	11
With family	16 (42)	9 (25)	36 (51)	15 (8)	14 (49)	34 (29)	15 (7)	21
Crèche	<1 (1)	0 (0)	5 (7)	19 (10)	0 (0)	0 (0)	17 (8)	6
Other*	71 (184)	54 (147)	59(85)	64 (33)	67 (228)	66 (57)	68 (32)	62
Children experience physical harm	74.3 (263)	82.1 (299)	85.4 (55)	49.4 (31)	68.3 (332)	57.6 (17)	20.5 (38)	72.2
Age of daughters entering sex work (years)	13.46 (267)	13.81 (268)	18.22 (52)	13.93 (29)	14.38 (289)	16.64 (37)	16.36 (39)	14.33
Daughters experience sexual abuse	76.8 (271)	84.5 (305)	48.8 (49)	41.8 (11)	70.1 (342)	40.0 (31)	13.6* (45)	74.0**
Sons experience sexual abuse	63.6 (267)	73.4 (307)	34.8 (40)	17.1 (7)	43.2 (337)	3.8 (13)		57.3**
Daughters enter sex	67.1 (270)	74.5 (298)	30.7 (109)	60.8 (49)	57.5 (328)	45.3 (47)	45.0 (50)	60.7
Age of daughters entering sex work (years)	13.46 (267)	13.81 (268)	18.22 (52)	13.93 (29)	14.38 (289)	16.64 (37)	16.36 (39)	14.33

In Brazil, daughters’ and sons’ questions were combined. Weighted average = Σ [(country average) *(country n)/Σ(country n)]. *Other = Brothels, on the street, with other sex workers, or at the mother’s job site (workplace). ** Excludes Brazil in the calculation of gender specific weighted average. *n* = total number of participants who answered each question (responses).

### Early childhood exposure: where FSWM leave their children while they work

The system of child development begins within the microsystem (Figure 1). The CFSW were left as infants in seemingly unprotected microenvironments in some study countries (Table 2).

The vast majority of FSWM left children in “other” locations, including high-risk locations like brothels, on the street, with other sex workers, or at the mother’s job site (workplace). In DRC, Angola, and Kenya, the second most frequent option was leaving the child alone at home. FSWM reported that the children were left alone while their mothers were at work due to a lack of services and affordable childcare resources. Study participants revealed that leaving children alone made them prone to disasters such as accidental fires, during which one FSWM indicated instances of child deaths due to burns. The second most frequent place that FSWM left children in South Africa and Brazil was at a *crèche* (daycare), while in Nigeria, Indonesia, and India, FSWM reported leaving children with a family member. In a few countries, including Indonesia, India, and Brazil, the zero responses in Table 3 indicate that children are not left alone. In Kenya, Indonesia, and the DRC, no responses indicated that children were left at a *crèche.* Apart from leaving CFSW with family, neighbors, coworkers, and a *crèche,* other microsystem environments provided unsafe exposures that compromised the children’s well-being.

**Table 3:** Environmental exposures of sons of FSWM with outcomes.

Exposure category	Code	Outcomes: Mothers’ comments
Poor living environments	Street	“Become street boys”. “Live on the street/slums,” “become thieves,” “Do not have a chance [unavailable in the environment] for education”.
	Forced to leave home	“Some sons run away, choose to live elsewhere”. “Move from house to house because of threats”
	No educational opportunities	“Drop out of school”. “Few get an education”. “None gets education”.
	Labor exploitation	“Rich people misuse our children”. “Sons become slaves”. “Start working at hard labor at age ten”.
	No work opportunities	“Do not get jobs because mom is a sex worker”.
Exposed to sex work	Exposed to sex work	“Some see mom with clients”, “Hang out at hotspots”.

### Negative outcomes experienced by sons and daughters

The estimates of negative outcomes (Table 2) were calculated based on the study participants’ answers to questions 2 (a, b) and 3 (a, b) listed in Table 1.

Across all countries, the majority of CFSW were reportedly sexually abused and physically harmed (Table 2). The FSWM report-based prevalence estimate of physical harm was high (country average 72.2%), ranging from a low of 20.5% in Brazil to a high of 85.4% in India. The second highest percentage of reported physical abuse was in the DRC (82.1%), and the third highest (74.3%) was in Nigeria (Table 2). Based on the estimates, daughters in DRC, Nigeria, and Kenya had higher percentages of sexual abuse (84.5, 76.8 and 70.1%, respectively) than sons (73.4, 63.6 and 43.2%). The lowest FSWM estimate of daughters’ and sons’ sexual abuse was in Indonesia (3.8%), possibly due to response bias since the data collection was carried out in the city of Jakarta, Indonesia, where the majority of FSWM came from villages. Reflection about the community knowledge revealed that Indonesian female sex workers usually return to the village after they get pregnant to give birth, and the children are raised in the villages by trustworthy family members when the mother works in the city. This may result in the lowest percentage.

According to participants, it is common for daughters of FSW to “enter sex work.” The estimated percentage, based on participants’ data, across all eight countries was 64% (Table 2), with individual country estimates ranging from 40% (lowest in Angola) to 74% (highest in India and Nigeria). The second-highest estimated percentage of entry into sex work for daughters was in DRC (67%). The average estimated age for FSW daughters “entering sex work” across all countries was 14 years, with individual country average ages ranging from 13 to 18 years (Table 2). While there was limited variation in average across countries, Nigeria, DRC, and South Africa had the lowest estimated age of “entry to sex work” at 13.4, 13.8, and 13.9 years, respectively. Kenya’s average age of entry was 14.3 years.

### What happened to the daughters of FSW?

Although gender-stratified qualitative text data analysis was not conducted to explore what happened to daughters, some noteworthy comments were provided. Participants reported that daughters of FSWM “enter sex work” at a young age (on average, 14 years) and in some instances were physically and sexually abused, as noted in Table 2. As the quantitative data suggest, some FSWM take young children to their workplace or bring clients home (62%, Table 2, where mothers leave children under the ‘other’ category). Bringing clients home exposes daughters to sex work in the home environment (microsystem).

One FSWM stated that mothers introduced their workplace to daughters, possibly as a future income source for them. As one mother stated, *‘Some sex workers…introduce their daughters to the hotspot.’* – Kisumu, Kenya.

Some others reported that their daughters followed their mothers’ path for economic survival.

*‘Daughters copy their mothers; the sex worker mom brings clients to the house, so the daughter sees what her mom does and copies her.’* – Kisumu, Kenya.

Daughters entering sex work were reported to be the result of mothers exposing daughters, at an early age, to their sex work environment. The text data provided explanations to the situation, indicating that the daughters get exposed to mothers’ work environment (Figure 1 mesosystem) and become subject to sexual abuse, as one study participant reported: *`The [sex worker] mother’s friends sexually abuse their* [FSW] *daughters.’* – Abuja, Nigeria.

In addition, since some daughters bear responsibility for caring for siblings at an early age, their only option to provide for their siblings after the mother’s death is sex work. In some situations, out of necessity, when mothers ceased work or died, their daughters entered sex work. As one mother reported:

*‘When a sex worker dies, her daughter enters sex work to care for her siblings; it is common.’* – Kisumu, Kenya.

Among other social and behavioral risks, their daughters’ entry into sex work is influenced by the microsystem home environment, which is shaped by mothers bringing clients home, and daughters taking on the responsibility of caring for siblings, as well as the mesosystem of mothers’ work environmental exposure.

### What happened to the sons of FSW?

Results of the textual analysis of what study participants reported as having happened to sex worker mothers’ sons, including a detailed analysis of social, behavioral, physical, and psychological vulnerable exposures and outcomes, are presented in this section. It is noteworthy to recall that the quantitative assessment by the respondents indicated that the majority of CFSW were subject to sexual abuse in countries like Nigeria (63.6%) and DRC (73.4%). However, the question was not asked in all study locations. The following text data analytical results provide an overview of what happened to sons, including bioecological environmental vulnerable exposures and physical and psychological outcomes.

**Figure 2:**
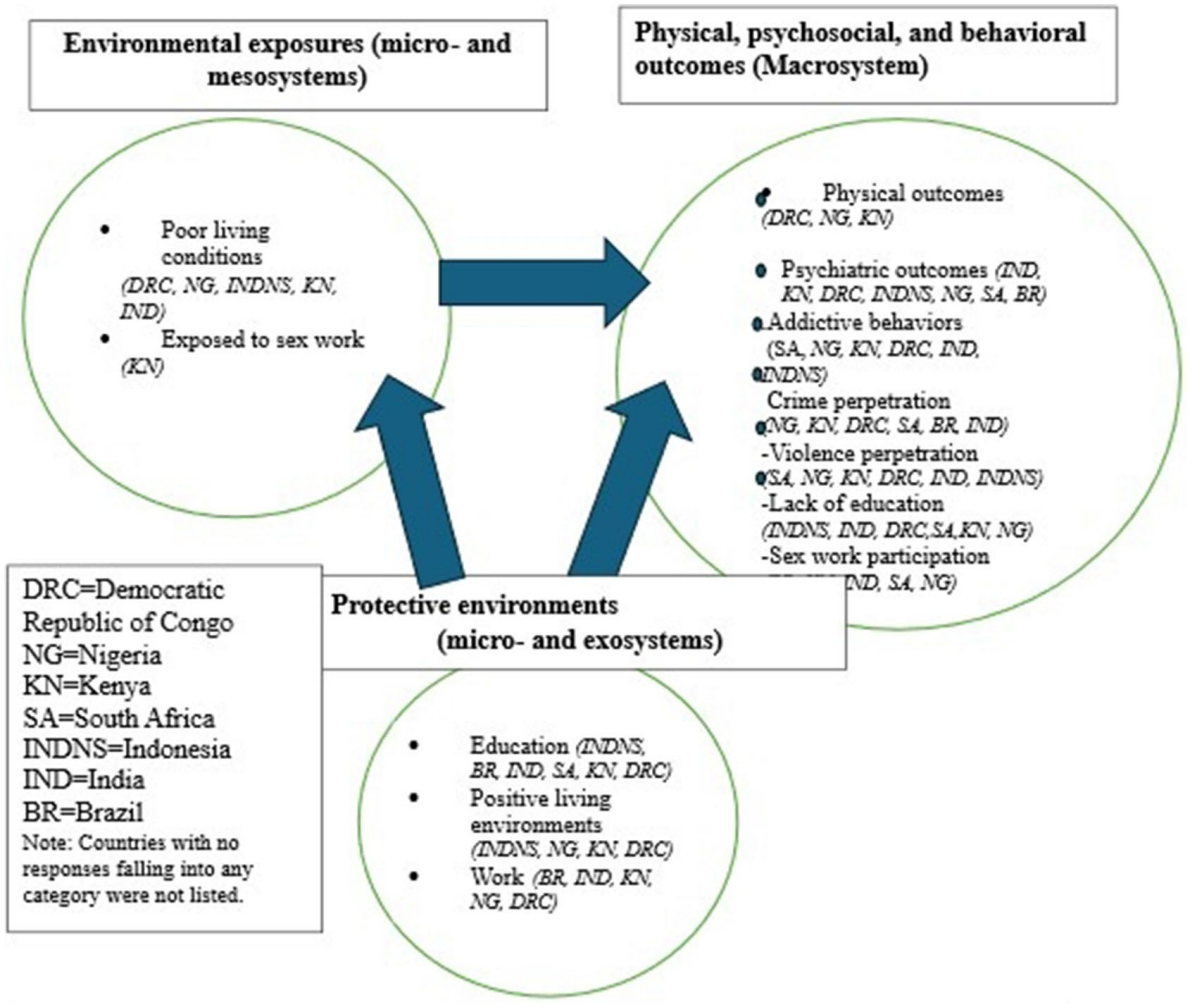
Coded categories of sons exposure and outcomes alignment with the working model.

A total of 429 study participants responded to the question, “What happens to sons of FSW in this community?” Free coding of the textual data resulted in the construction of three major categories: (a) environmental exposures, (b) physical, psychosocial, and behavioral outcomes, and (c) protective factors that may mitigate adverse outcomes. Figure 2 illustrates the respective categories and related codes, along with their distribution across countries. Although free coding was conducted, the research team applied the working model (Figure 1) to interpret the results, which are summarized in Figure 2. Arrows indicate exposures and outcomes, illustrating how positive environments can mitigate negative ones.

### Environmental exposures

Sons are exposed to social and behavioral challenges through vulnerable exposure to violence and sex work through their mothers’ occupations and poor living environments (Table 3), as noted in the micro- and mesosystems (Figure 1). Some stated that their FSW friends (peers in Figure 1, microsystem) and clients sexually abused their sons (on average, 57.3%; Table 2 shows the percentage distribution). Violence experienced in sex work and community environments (exosystem) was reported in three African countries – DRC, Nigeria, and Kenya (Table 2). This violence also occurred to a lesser extent in India and Indonesia.

Poor living environments (mesosystem, Figure 1) included living on the street and depriving sons of opportunities for education or stable employment. These environmental exposures were noted in the text data, mostly in the DRC, followed by Nigeria and Indonesia, with the lowest reported occurrences in Kenya and India. Impoverished and unhealthy living situations, as well as their associated interactions, were reported more frequently in the DRC than in other countries. Nigeria had the second-highest number of such text responses. The following direct quotes provide further context to the situation. Some in Kinshasa, Nigeria, stated there is “no home” for children to live in; thus, they become street kids.

‘*The majority become bandits, street children, because they see the way the mother is living*’, Cape Town, DRC.

‘*We have to move from house to house because of threats to us and our children*.’ – Nairobi, Kenya.

Some mothers reported that their sons beg and survive off the streets or end up going to prison:

*‘Some [sons] eat from the trash cans.’* – Calabar, Nigeria.

They also cited a general lack of work opportunities for sons, in some cases due to stigma and discrimination, a set of macrosystem factors resulting from the mother’s occupation:

*‘Some do not get jobs because mom is a sex worker.’* – Salem, India.

Labor exploitation of sons was also reported, with sons entering manual labor at young ages and being mistreated or becoming “slaves,” as two mothers in Kenya expressed:

‘*[Sons] start working at hard labor at age ten*.’ – Kisumu, Kenya.

‘*[Sons] become slaves.’* – Mombasa, Kenya.

*‘Some see mom with clients and* [when sons] *hang out at the hotspots.’* – Mombasa, Kenya.

Kenya was the only country where FSWM reported that sons had direct exposure to their mother’s occupational environment (sex work). However, evidence from other categories—such as sons becoming sex workers or engaging in relationships with sex workers—suggests that direct micro-level exposure likely occurred but was not explicitly reported in the other study countries. It is possible that sons are exposed to sex work when they hang out at hotspots, and some even observe their mothers with clients at a young age, as noted in Table 2. The impact of this microsystem factor may be more severe for this population because of the mothers’ high-risk occupational exposure introduced at such a young age.

### Physical and psychosocial outcomes

The next major category was sons’ physical, psychosocial, and behavioral outcomes (Table 4) and included seven subcategories. Physical outcomes reported included physical harm, including injury from gunshots, being murdered by mobs, and sexual abuse. As one mother observed,

**Table 4:** Physical, Psychosocial, and behavioral outcomes of sons of FSWM.

Category	Code	Mothers’ comments
Physical outcomes	Sexual abuse	“Mom’s friends sexually abuse sons”. “Mom’s clients sexually abuse [their sons]”.
	Physical violence	“Some get shot”.
	Murder	“Get killed by mobs”.
Adverse psychosocial outcomes	Abuse mother	“They become abusive to their moms and are angry [at them] for leaving them”.
	Become angry	“They become angry when they learn their mom is a sex worker”.
	Develop a negative character	“Lack of respect for society”. “Some become mentally disturbed”.
	Experience discrimination	“Discriminated against”.
	Become judgmental	“Do not respect mom, because mom is a sex worker”.
	Join cults	“Join a cult”.
Perpetrate crime	Become criminals	“Once a boy knows his mom is a sex worker, he may commit crimes”.
	Become drug dealers	“Start drug abuse because they join gangs, because mom is not around”.
	Become gangsters	“Become gangsters, bandits, thugs”.
	Become robbers	“Become bandits, thieves, robbers, pickpockets, muggers/thugs”.
	Become terrorists	“Become homosexuals, thieves, drunkards, terrorists”.
	Become child and drug traffickers	“[Sons] pick up children and sell them”. “Some become drug traffickers, use children to transport drugs”.
Substance and alcohol use	Take drugs	“At age 10, they start using drugs and fighting”.
	Become alcoholics	“[Sons] learn mom is a sex worker and start to drink, become alcoholics”.
	Gamble	“[Sons] do gamble”.
Perpetrate violence	Fight	“Fight in the street with peers and other kids”.
	Murder	“[They] Kill”.
	Rape	“[Sons] become rapists and rape street girls”.
No education	Drop out of school	“They leave school because they are stigmatized”.
	Become uneducated	“Not well educated”. “None gets education”.
Sex Work	Become sex workers	“Start to work as a sex worker or pimp”. “Peer pressure to enter sex work”.
	Have sex with sex workers	“Take drugs and have relationships with sex workers”.
	Have sex with sugar mama*	“Sons look for sugar mama”. “Marry older women”.

*Older women who have transactional sex with young boys.

“*Most become like people without a conscious because of what they have seen, and [being] raped by others.*” Kinshasa, DRC.

The second subcategory, comprising adverse psychological outcomes, was reported by FSWM in most countries, except Indonesia, with more comments from India and the fewest mentions in Brazil. Some CFSWs are angry and abuse their sex worker mothers, perhaps because of their occupation. The third subcategory was the perpetration of crime and violence by sons, reported in all countries except Indonesia, with the most reported occurrences by Nigerian study participants, followed by Kenyan, Congolese, and South African FSWM. This may be because most CFSWs live in villages away from their mothers’ work environment.

The fourth subcategory related to drug, alcohol, and other substance use, mostly reported in the African countries (South Africa, Nigeria, Kenya, and DRC) and to a lesser extent in India and Indonesia. The fifth subcategory relates to CFSW perpetrating violence, such as fighting with other children, even murdering and raping them, and FSWM reported this in every country except Brazil. The sixth subcategory, which described dropping out of school and becoming sex workers or pimps themselves, was reported everywhere but Brazil. The differences noted in Indonesia may be underestimated due to the small sample size originating from the city of Jakarta.

The research team noted other consequences reported by FSWM, such as sons being abusive, angry, or judgmental toward their mothers and others due to the negative stigma associated with sex work, including discrimination at schools and a general lack of respect from society. These other social, psychological, and behavioral consequences occur in the macrosystem depicted in Figure 1.

*‘[They] do crime, raping, robbery, go to jail, use drugs.’* – Cape Town, South Africa.

*‘They become abusive toward their moms and are angry [at them] for leaving them.’* – Durban, South Africa.

*‘They become angry when they learn their mom is a sex worker.’* – Nashik, India.

*‘Some get angry and fight with other kids.’* – Jakarta, Indonesia.

As one mother reported, *‘At age ten, they start using drugs and fighting.’* – Calabar, Nigeria.

Substances used included alcohol, marijuana, glue, and other illegal drugs. The crimes reported varied in degree from theft, robbery, and human and drug trafficking to rape and murder. It should be noted that the highest percentage of mothers in DRC reported that their children were experiencing physical harm (82.1%). In the text data, one mother reported that the majority, like 90% become thieves and bandits, thus becoming vulnerable in the legal system.

*‘[Sons] become alcoholics.*’ – Calabar, Nigeria.

*‘The majority [of sons] become thieves and bandits, like 90 %.’* – Bukavu, DRC.

*‘Some become drug traffickers [and] people use* [sex workers’] *children to transport drugs.’* – Mombasa, Kenya.

Some FSWMs expressed that their sons were drawn to such behaviors due to the lack of a mother’s presence in their lives and their low self-esteem, both of which they attributed to having a mother who is a sex worker, who deprives sons of cognitive stimulation.

*‘Because there is no parental guidance, they join gangs.’* – Kisumu, Kenya.

Another reason noted was the influence of mothers’ work environment, where they were exposed to violent behavior.

*‘They are violent, because they see clients beat their mothers.’* – Calabar, Nigeria.

*‘They become violent toward everyone.’* – Johannesburg, South Africa.

FSWM reported developmental and cognitive challenges. It was common for their sons to drop out of school (microsystem), sometimes due to the stigma associated with being CFSW, while others did not receive an education at all. The highest number of responses regarding a lack of education for sons came from Indonesia and India.

*‘They leave school because they are stigmatized.’* – Bukavu, DRC.

*‘*[Only] *Few [sons] get education.’* – Nashik, India.

Finally, many participants commented that their sons end up becoming sex workers or pimps themselves.

*‘Some join mom as a sex worker.’* – Johannesburg, South Africa.

*‘They become pimps because they think it is an easy life.’* – Rio, Brazil.

Some reported that sons have sexual relationships with sex workers in the community or seek out relationships with “sugar mamas” – older women who have transactional sex with young boys.

*‘Some look for sugar mommy.’* – Kisumu, Kenya.

Brazil had a substantially higher number of study participants who described sons becoming sex workers or engaging in transactional sex with “sugar mamas” (older women who seek sex from young men) and sex workers, compared to the other countries. In addition to the above psychological outcomes, FSWM in India noted negative societal impacts on them, as one mother said, “*They get teased.” In* Kenya, mothers indicated that “*they lack respect in society,*” which resulted in poor psychological outcomes, such as having low self-esteem and living in regret.

### Protective environments

Participants reported protective factors that prevented negative bioecological outcomes. This category encompassed various situational and environmental-related codes that positively influenced psychosocial and behavioral outcomes, as well as the general well-being of the sons of FSWs. Table 5 lists these protective factors, derived from the study participants’ responses, including education, formal and informal work, and other positive living conditions (belonging to Figure 1 microsystems) that, according to participants, contributed to the sons ‘doing okay.’

**Table 5:** Protective factors that influenced physical, psychosocial, and behavioral outcomes for sons.

Category	Code	Mothers’ comments (example)
Education	Receive education	“sends son to boarding school”. “One or two do okay, because they study”.
Positive living conditions	Church	“(If they) get help from churches, (they) do okay”.
	Live elsewhere	“They do okay if they live with other family members”.
	Positive parenting	“They need a good role model; (if they) go bad depends on the parents”.
	Not knowing that mothers are sex workers	“[If] sons do not know mom is a sex worker”
Work	Informal work	They “Become garbage collectors”. “Become hawkers”. “Sell scrap materials”.
	Formal work	They “Get white collar jobs”. “Sons work to get [their] mothers out of that life”.

Education was noted as a protective factor for the adverse outcomes reported above. According to participants, receiving an education, attending school, and even sending sons to boarding schools mitigated some of the negative impacts of exposure to sex work. Aligning with the bioecological model (Figure 1), these protective factors fall within the microsystem.

It was reported that younger FSWM protect their children by removing them from a vulnerable living environment.

“*If mom is young mom, (she) sends (her) son to a boarding school*.” -Cape Town, Kenya.

*‘One or two [sons] do okay, because they study.’* – Bukavu, DRC.

Additionally, positive attributes of sons’ living environments—such as having a strong church community, sons living with other family members, or the mothers’ ability to be a positive role model—were described as protecting sons from the adverse impacts of being raised by an FMSW. These protective factors were reported in Indonesia, Nigeria, Kenya, and the DRC only, with mothers from Indonesia most frequently citing such positive living conditions. It is worth noting that FSWM in Indonesia reported that they generally give birth in the village and then leave their children with relatives when they return to work in Jakarta. In contrast, many of the sons of FSWM in other countries live with their mothers. The importance of mothers’ influence on their cognitive development was emphasized.

*‘How (do) some do okay? It depends on the effect of the mother. They also do okay if they live with other family members.’* – Bukavu, DRC.

*‘You [the mother] must be a good role model. What sons do depend on the mother’* – Mombasa, Kenya.

Some FSWM spoke about sons not knowing about their mother’s work as a protective factor:

*‘[If] sons do not know (his) mom is a sex worker, [there’s] no problem.’* – Jakarta, Indonesia.

Finally, some FSWM mentioned that sons who worked at jobs did well, with the highest number of such responses from India and Brazil. Work included formal jobs, such as ‘white collar jobs,’ as well as informal work, including ‘hawking’ and ‘garbage collecting.’

*‘The son usually thinks of working to take his mother out of that life.’* – Rio, Brazil.

*‘They go to work in factories.’* – Nashik, India.

Although schools are considered protective environments within the mesosystem noted earlier, FSW children have faced stigma in schools, leading to their dropout and ultimately depriving them of educational opportunities (Table 3).

Responses collected from FSWM were described using the working model (depicted in Figure 1), which was developed based on the revised bioecological model (22). The results aligned with applications of the model in Figure 2, revealing common and unique social and ecological environmental exposures within which CFSW were raised and lived (Figure 2). The unique bioecological environment was determined by living with an FSWM as her child and being exposed to her occupational environment. These were major influential factors for the negative physical and psychological outcomes observed in early adolescence.

From the life course perspective, children’s trajectories of exposure contribute to their negative developmental outcomes. This starts with their early childhood exposures in family and work environments at the microsystem level, such as being left alone at home or their mothers’ work, followed by sex work-associated social environments in the mesosystem and exosystem (such as unstable housing and street living), and finally, their exposure as grown-up children facing labor exploitation and societal discrimination in the macrosystem—all of which contribute negatively. However, educational, positive living, and work environments (Table 5), which expand from the microsystem to the outermost macrosystem, are seen as providing protective environments that promote positive developmental outcomes.

## Discussion

This study documented the bioecological vulnerability exposures, physical, psychosocial, and behavioral outcomes, and the protective environments of CFSW, based on data collected from FSWM about their children and other CFSW living in the community. Bioecological exposures and outcomes were contextualized using a working model adapted from the revised Bronfenbrenner’s *Ecological Model of Human Development* (22) as the theoretical framework (Figure 1). This study is partially connected to the theory of human development (26) because the model is applied only in the interpretation of results stage of the study, not in the study design, analysis, or coding stages. This study is the second to apply Bronfenbrenner’s *Ecological Model of Human Development* to CFSW. The first study applied the theory to brothel-based children living in Mumbai, India, by studying the environments in the microsystem, mesosystem, and exosystem (19). The current study’s findings, from eight LMICs, included results belonging to all four systems, including exposures to the macrosystem. It also looks at stigma and discrimination experienced by CFSW living in the community and includes protective environments (Figure 2), which were not included in the single-city, brothel-based Indian children study (19). The bioecological environmental exposures of this study were not limited to brothels.

Our study showed that CFSW are vulnerable to unsafe environmental exposures that began in early childhood and resulted in negative developmental outcomes. Reported outcomes varied in frequency by country and region across eight LMICs. Adding validity to our findings, a prior study in Uganda found that CFSW living in sex work communities experienced violence from family members and relatives, including the mother, all of whom are in the microenvironment.

Our FSWM report-based estimates suggest that the majority of CFSW have been physically harmed and sexually abused, which is a much higher percentage than reported by UNICEF (27). UNICEF acknowledged the issue of the abuse of girls in DRC, even with limited data (28), indicating that 12% of children in Eastern and Southern Africa (between 15 and 19 years old) were physically abused, mostly by people known to them, such as family members or friends. This percentage was three times higher for boys in most African countries (27). Physical and psychological outcomes observed for CFSW in the current study, in the African region, were twice the regional estimates. These additional vulnerabilities are preventable if safer growing environments (Figure 2) and expanded micro-, meso-, and exosystems (Figure 1) are ensured.

Our study estimated a high percentage of daughters of FSWM entering sex work at a young age, as young as 13. Based on FSWM reports, the average age of daughters entering sex work across the eight countries was 14 years. The young age of CFSW daughters entering sex work (13–14 years) in African countries is particularly alarming (Table 2). A study conducted in Nairobi, Kenya, reported that a common pathway into underage sex work was early childhood sexual debut, with entry into sex work as young as 15 years, which is consistent with our findings (28).

The current study suggests that nearly two-thirds of young children of FSWM are left in vulnerable environments, including alone at home, on the street, or where mothers meet clients. This finding is validated by a previous study’s findings from a low-income resettlement neighborhood in Nairobi, Kenya (one of our study cities), where one in five mothers reported leaving their children (0–2 years of age) alone for more than one hour (29). A study conducted in South Africa found that seven out of 18 children studied were taken to the mothers’ workplace (30). Though this may be a common practice in some countries, workplaces of FSWM are typically brothels or hotspots, with additional risks imposed on CFSW’s well-being and healthy psychological and physical development. An additional study conducted in Kenya further validates the current study’s findings that exposure to sex work harms CFSW’s psychological well-being (16). The present study found that FSWM childcare practices follow the pattern of behavior of mothers in the country and/or region; however, the severity of CFSW exposure to harmful environments is alarming, requiring further investigation in future studies. The exposures and outcomes uncovered through quantitative analysis were further validated and contextualized using qualitative analysis, as discussed below.

Early childhood exposure to sex work occurred when some mothers introduced their daughters into hotspots, their workplace, at an early age. Some daughters entered sex work to take care of siblings after their mother’s death. Economic and social factors interact with family, community, and interpersonal factors to influence the sexual behavior of adolescent girls living in vulnerable situations (31). This intersection, illustrated in Figure 1 with two-sided arrows crossing in the mesosystem, suggests that FSWM may be creating high-risk environmental clusters at the intersection of multiple factors that influence adolescent girls’ engagement in risky physical, sexual, and behavioral activities. These findings need to be confirmed in a larger study that examines and validates direct exposures and related outcomes.

The microsystem-level vulnerable exposures (Figure 2) for sons include living in brothels, living alone, and witnessing physical and sexual abuse and harm against their mothers and other sex worker women. Sons’ begging for food on the street was another vulnerable exposure to age-inappropriate sexual relationships, physical and sexual abuse, and labor exploitation. Other risky behaviors noted for sons include drug and alcohol abuse, gang involvement, and perpetration of violence and crime. Research suggests that children in the juvenile system were exposed to adverse environmental factors and were involved in the noted high-risk behaviors prior to entering the system (32). At the macrosystem level, social and cultural norms, stigma, discrimination, and societal ideologies associated with being a CFSW add another layer of influence on sons’ adverse outcomes: seeking out a ‘sugar mama’ or turning to gambling, alcohol use, and other socially unacceptable behaviors. CFSW identity-associated stigma and discrimination influenced CFSW to drop out of school, have trouble finding work, frequently shift residences due to threats, and become victims of violence and murder. A study among minority youth in the US found greater acts of violence experienced by stigmatized or minority populations (33), and the situation of CFSW in LMIC is likely to be even worse.

CFSW vulnerable exposures can be minimized by raising them in safe environments (Figure 2, Table 5) and expanding multiple layers that cover micro-, meso-, and exosystems (Figure 1). It is paramount that CFSW should be protected from, or removed from, these unsafe environments to prevent them from being involved in criminal activities or becoming victims of violence and crime. Good education, attending school, living away from the FSWM work environment, and having work opportunities can protect CFSW from the aforementioned vulnerabilities and dangerous bioecological exposures, as well as negative outcomes. Study participants viewed education and employment opportunities for children as a means for CFSW to escape sex work. Education and work also protected CFSW from becoming part of the intergenerational sex industry prevalent in countries like India (19). Other protective factors identified by the research participants included positive parenting and mothers serving as positive role models for their children, formal or informal work opportunities for children, supportive social networks such as church communities, and access to quality childcare services. Family-centered care was superior to being raised by untrustworthy family members in a study of CFSW and drug users in Zambia (1). Dalla et al. described a center established in India by the NGO *Inspire* that provides brothel-based children with a safe, overnight care environment while their mothers work (19).

The present study’s findings suggest that microsystemic vulnerabilities in sex work occupations interact with the exosystemic sex work environment (as shown in Figure 1, with intersecting arrows) to create some of the ecological vulnerabilities for CFSW. The political and economic landscapes of a child’s societal context exert influence on child exploitation. Broader public health and child welfare approaches are needed to reduce children’s vulnerability to victimization (34), to create economic opportunities, and facilitate upward social mobility (35). These exosystemic-level protective factors warrant further exploration in future studies.

Although triangulation of prevalence estimates based on reports from hidden populations is challenging (1), reporting bias may be influenced by the stigma associated with these populations. However, a meta-analysis revealed that research conducted by independent teams, such as the present study team, may help reduce reporting bias (36). The findings of this study are supported by existing literature on the vulnerabilities of CFSW. In particular, the CFSW vulnerabilities we identified—such as becoming victims of abuse and/or violence and/or leaving home and becoming sex workers themselves—were also reported in a study from Iran that used data from FSWM (37). Moreover, our findings on stigma, discrimination, and the denial of equitable access to housing and education parallel the experiences of CDSW in Bangladesh (38). Although the present study did not collect data directly from CFSW, the perspectives of FSWM carry increased validity because they align with findings from similar studies, including a review of nine published studies on CFSW(4).

## Limitations and future directions

When data are collected in a group setting, there is a potential for response bias, especially when participants may respond in a way perceived to be favorable to the interviewer, who may be from an NGO providing support services, and/or in a way that is influenced by other participants’ responses. The study participants’ comments on what happened to sons and daughters were brief and therefore did not warrant thematic analysis using narratives, nor did they allow for triangulation of text data to mitigate this bias. Validation of mothers’ reports of children’s development and well-being using individual data collection from the hidden population of CFSW may not be feasible to mitigate the response bias due to their age and vulnerability. The application of Bronfenbrenner’s theoretical framework (23) was limited to the data analysis and interpretation of results stage, rather than the data collection stage, which would have enhanced the findings presented across bioecological exposures.

Though previous studies among this population reported child deaths, filicide, and mothers using drugs and alcohol during pregnancy, potential harm to infants and young children who are exposed to dangers (25), this study did not explore the situation of orphaned CFSW and any developmental problems among CFSW due to in utero drug and alcohol exposure. In addition, while mothers reported many problems among CFSW, the research team did not probe them with specific mental health issues such as depression, suicide ideation, or attempted suicide. Even further, this exploratory study’s data collection was limited to reports from the mothers engaged in sex work, and no data were collected directly from other family members or children of sex workers, nor from those who have left sex work. This reporting bias may reduce external validity.

Further research, employing a longitudinal life course, is necessary to investigate risk and protective factors for sons and daughters of FSW across all age groups, providing concrete evidence on developmental outcomes and informing the development of mitigation strategies.

## Conclusion

This is the first multi-country study to explore the common effects of the sex work environment on children raised by FSWM. The findings illustrate how the bioecological vulnerabilities of mothers’ sex work impact the physical, psychosocial, and behavioral outcomes of CFSW. Most of the findings are consistent with the research-based evidence on CFSW from single-country, single-city, and multi-site review studies. This affirms the research team’s use of an exploratory study model, as well as the data collection instruments and settings, all of which can be applied to future studies of this hard-to-reach population.

Results indicated that CFSW in LMICs experience multiple threats to their physical and psychological developmental outcomes and social well-being. Lack of proper child-rearing practices and exposure of children to high-risk environments exacerbate their vulnerabilities. The CFSW studied experience risks at every level of their ecosystem, as depicted in the human development paradigm, including within the family and community, as well as in distal social environments. These hazardous environmental exposures jeopardize the future development of these children.

On an individual level, these harmful exposures cause lifelong physical, emotional, and mental health problems. On a community and societal level, they undermine efforts to achieve the United Nations 2030 Sustainable Development Goals (SDGs) of ensuring good health and well-being (SDG 3) for all (39). The study findings highlight the urgent need for evidence-based programs and support for vulnerable FSWM and their children, as well as funding to implement and sustain these programs.

## Data Availability

The raw data supporting the conclusions of this article will be made available by the authors, without undue reservation.
